# Near-Infrared Spectroscopy Monitoring in Pediatric Anesthesiology: A Pro-Con Discussion

**DOI:** 10.7759/cureus.13875

**Published:** 2021-03-14

**Authors:** Anusha Rao, Bharathi Gourkanti, Noud Van Helmond

**Affiliations:** 1 Anesthesiology, West Virginia University, Morgantown, USA; 2 Anesthesiology, Cooper University Hospital, Camden, USA

**Keywords:** anesthesiology, nirs, near-infrared spectroscopy, pediatric anesthesiology

## Abstract

Near-infrared spectroscopy (NIRS) has been increasingly used as a non-invasive measurement of cerebral tissue oxygen saturation. The aim of this short review is to discuss the benefits and drawbacks of its use in the pediatric anesthesia population. In the context of cardiac surgery, lower intraoperative NIRS values have shown a modest association with neurodevelopmental outcomes while lower neonatal intensive care unit NIRS values have been correlated with reduced neurodevelopment in children. However, it is still unclear if management aimed at increasing cerebral tissue oxygenation would have any benefit on these outcomes. Without prospective research looking into the effects of intervention given proper thresholds, the true benefit of NIRS use is still up for debate. Even with current research gaps, its use in the clinical setting continues.

## Introduction and background

Infants are vulnerable to hemodynamic instability, and brain injuries are an important underlying cause of long-term neurodevelopmental disabilities after surgery early in life. Near-infrared spectroscopy (NIRS) allows for the noninvasive monitoring of mixed venous oxygen saturation of hemoglobin in brain tissue and has garnered substantial interest among specialists in neonatal intensive care unit settings [1] and pediatric cardiac surgery [2] while also gaining traction in other types of pediatric surgery [3-4]. The premise of NIRS-derived monitoring of cerebral oxygenation and perfusion is that it may be possible to timely identify and intervene during episodes of suboptimal oxygenation and perfusion of the immature brain.

The aim of the present focused review is to describe the principles underlying NIRS, to present the current controversies on the use of perioperative and intraoperative cerebral NIRS monitoring, to identify current research gaps, and to identify potential future developments in the field.

## Review

Principle of near-infrared spectroscopy

**Figure 1:**
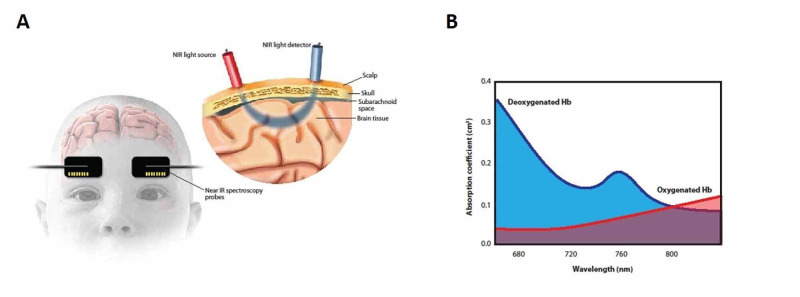
1A) Near-Infrared light source and detector. Photon penetration is dependent on the distance between the source and the detector; deeper penetration can be achieved with greater spacing. 1B) The different absorption wavelengths for oxy- and deoxy-hemoglobin.

NIRS makes use of the Beer-Lambert law, which states that the absorption of light of a given wavelength passing through a non-absorbing solvent, which contains an absorbing solute, is proportional to the product of the solute concentration, the light path length, and an extinction coefficient. In clinical applications aimed at measuring the oxygen saturation of hemoglobin, absorbance is usually measured at two to four different wavelengths, to detect both oxyhemoglobin and deoxyhemoglobin, as well as to overcome the influence of pulsatile flow and other tissues. NIRS probes are placed on the forehead, and they transmit infrared light (wavelength range 660-940 nm) that passes through skin and bone to the tissue (Figure 1A). Light detectors within the probe measure light that is scattered from the underlying tissue. By measuring the absorption in the specific range for oxy- and deoxyhemoglobin (Figure 1B), NIRS devices are able to calculate the regional tissue oxygenation (rSo2) in the underlying tissue. Because the sensors are placed on the skin, the depth of penetration in the cortex is dependent on the thickness of tissue in the light path that needs to be traversed before getting to the cortex. In both adults and neonates, light penetrates 25-30 mm into the head, measured from the surface of the scalp. Neonates have a thinner skull and skin than adults and light penetration into the cortex is 10-15 mm in neonates, whereas it is only 3-5 mm in adults [5]. Normal values for cerebral oxygen saturation in the neonatal brain can range from 55% to 85% [6]. The cerebral vasculature volume is approximately 75% venous and 25% arterial, and the NIRS measurement thus reflects a larger proportion of venous saturation. Absolute NIRS values can be influenced by sensor pad placement, gestational age of the infant, skin pigmentation [7], and the type of NIRS device used [8]. Additionally, increased conjugated bilirubin concentrations have been shown to lower absolute NIRS values in neonates [9] and adults [10].

Arguments pro using NIRS in pediatric anesthesia patients

Physiology and Validity of Measurement

Proponents of using NIRS in pediatric anesthesia point out that NIRS provides a unique, non-invasive opportunity to measure brain oxygenation in a population that may otherwise be difficult to monitor due to hypoplastic and small vessels. Additionally, central hemodynamic measurements may not reflect cerebral perfusion since the brain’s oxygen supply is relatively independent due to autoregulation*. *Cerebral autoregulation refers to the maintenance of a relatively constant cerebral blood flow over a broad range of perfusion pressures, the so-called “autoregulatory plateau.” The autoregulatory plateau in the adult human operates over mean blood pressures ranging from ∼60 to 150 mmHg [11]. Autoregulation is functional at approximately 36 weeks of human gestation and has been shown to improve progressively between 23 and 33 weeks of gestation in premature neonates [12].Moreover, even reliable indicators of cerebral blood flow, such as transcranial Doppler ultrasonography, do not provide a measure of oxygen extraction and may not always be related to cerebral ischemia [2]. When considering the clinical setting, it is more difficult to obtain a signal with transcranial doppler ultrasonography and to prevent probe dislodgement than to apply NIRS probes to the forehead [13]. Because there is no gold standard measurement of tissue oxyhemoglobin content, jugular venous oximetry has often been used as a standard to compare NIRS to. Both preclinical [14] and clinical investigations [15] have found strong correlations between NIRS and jugular venous oximetry.

Relationship With Clinical Outcomes

Most studies linking perioperative NIRS measurements to clinical outcomes have been in the context of cardiac surgery. Low cerebral oximetry values have been found to be correlated with mortality and major postoperative morbidity, including prolonged intensive care stay, prolonged mechanical ventilation, and the need for extracorporeal membrane oxygenation [2]. This has been demonstrated after the Norwood procedure (a three-stage heart surgery to create a functional systemic circuit in patients with hypoplastic left heart syndrome) and in mixed pediatric cardiac surgery cohorts in which cyanotic and noncyanotic patients were analyzed together [16-17]. NIRS measurements appear to be accurate both in noncyanotic and cyanotic patients [18]. Among infants with hypoplastic left heart syndrome perioperative cerebral oximetry values, along with several demographic and disease-related factors, correlated with neurodevelopmental performance at preschool age [19]. Visual-motor integration scores, used as a form of neurodevelopmental assessment, were less than the population mean in the patients with hypoplastic left heart syndrome, and low NIRS values in the post-stage 1 palliation of the Norwood procedure were a risk factor for reduced neurodevelopment in childhood.

Reduced perioperative variability in NIRS values is also believed to be a sign of impaired cerebral vascular autoregulation and has been found to be associated with poor neurodevelopmental outcomes at 21 months of age in a mixed population of neonatal cardiac surgery patients [20]. Neurodevelopmental assessment in that study consisted of the Bayley scales of infant development, which is a standard series of developmental play tasks that derive developmental quotient scores in different domains that can be compared to norm scores obtained from typically developing children of the same age [21].

Goal-Directed Therapy

Based on correlations between perioperative NIRS measurements and the short- and long-term outcomes of surgery, cerebral oximetry values have been used as a target for support of oxygen delivery to the brain, similar to strategies for shock, collectively termed ‘goal-directed therapy.’ In a non-randomized observational study in a mixed pediatric cardiac surgery population, Austin et al. assessed the potential benefit of interventions based on intraoperative cerebral NIRS monitoring in decreasing in-hospital postoperative neurological complications such as seizures, cerebral infarct, chorea, visual neglect, hypotonia, and speech problems [22]. In a reference period during which NIRS values were collected, but not acted on, postoperative neurologic complications were detected in 26% of patients who had a monitoring event. After implementing a policy to act upon regional tissue oxygenation (rSo2) changes, neurological complications were seen in 6% of patients who had a neurological monitoring event. The 6% rate of complications was similar to the rate of complications in patients who had no monitoring events (7%). A monitoring event was defined as an at least three-minute decrease in rSo2 of >20% as compared to a patient's baseline before aortic cannulation. Similarly, a study in adults undergoing endarterectomy found that within-patient changes of <20% in rSo2 were unlikely to have neurological complications, whereas patients with a >20% in rSo2 had a higher chance of neurological complications [23]. Along these lines, proponents of NIRS monitoring have particularly advocated for the use of NIRS monitoring as a 'trend monitor' to follow changes within a patient, rather than acting on absolute rSo2 thresholds. 

Arguments con using NIRS in pediatric patients

Clinical Relevance

Opponents of NIRS point out that when looking at relationships between perioperative cerebral NIRS values and neurodevelopmental outcomes in different types of pediatric cardiac surgery, the correlation coefficients are typically modest. A study looking at infants one year after they had undergone biventricular repair without aortic arch reconstruction found a modest association (r = 0.23) between lower psychomotor developmental index (PDI) scores on the Bayley scales and a rSo2 <45% during different perioperative stages [24]. Moreover, even if the relationships were stronger, it is presently unclear if treatment based on abnormal NIRS values is associated with better outcomes since almost all studies to date are observational in nature.

Potential Risks of Intervention

The reliance on cerebral oximetry in conjunction with assumed ‘normal values’ may lead to unnecessary and potentially harmful overtreatment, possibly leading to hyperoxygenation, which is particularly dangerous due to the underdeveloped antioxidant defense system of infants [1]. This may be especially true for clinically stable patients with severe cyanosis, in whom the determination of normal values is particularly challenging [25]. Increasing the mean arterial pressure in small heparinized neonates with immature brains can result in intracranial hemorrhage. It also appears to be difficult to define proper thresholds in the intraoperative period, for example, with different bypass conditions. In this context, the early adoption of NIRS has been compared to fetal heart rate monitoring, which has led to an increase in obstetrical interventions without an appreciable decrease in long-term neurological complications of birth asphyxia [26].

It has been hard to develop proper thresholds for intervention in pediatric anesthesiology because of the variability of ‘normal’ cerebral oxygenation levels that exist in patients with, for e.g., congenital heart disease. Because of this variability that exists across the continuum of the perioperative period, it is difficult to determine one optimal threshold for rSO2. The thresholds may have to be different for the intraoperative and postoperative periods, as the intraoperative period requires a higher level of neuroprotection [1].

Cost

NIRS devices (CerOx - Ornim Medical, Israel; EQUANOX - Nonin, The Netherlands; FORE-SIGHT - CasMed, US; INVOS - Somanetics, USA; NIRO - Hamamatsu, Japan; OxyMon - Artinis, The Netherlands) are relatively expensive (prices range from $10.000 to $20.000) and individual sensors cost around $100 per patient. In the absence of definitive evidence supporting their use, opponents feel these costs are not justified. Additionally, different devices have varying degrees of capabilities; for example, NIRO, in addition to displaying saturation values, has the ability to detect blood concentration and concentration changes that occur in oxyhemoglobin, deoxyhemoglobin, and total hemoglobin. The technology of these devices continues to evolve and with future developments, it could be possible to have devices that detect multisite cerebral oxygen saturation. This could aid in having more insightful NIRS measurements to base treatment protocols on in the future.

Current research gaps

There is currently no randomized clinical trial evidence to support interventions based on inadequate cerebral NIRS values. It is important to note that this gap is not unique to cerebral NIRS monitoring; other very commonly used monitoring devices, such as pulse oximetry, have never been demonstrated in a randomized controlled trial to affect the outcome of anesthesia for patients [27]. The difficulty of reaching ‘level A’ evidence for monitoring devices appears to be related to the multiple steps that are required to obtain such proof: 1. A monitor technique needs to be valid and accurate, 2. Normal values need to be defined, and 3. Interventions based on abnormal values need to lead to improved long-term outcomes. Since decades of development and research may separate steps 1 and 3, it is not uncommon that monitoring devices become ‘standard of care’ before research reaches step 3, especially if the measurements are non-invasive in nature. Once a device has been adopted widely, it becomes increasingly challenging to conduct a randomized controlled study to address the ultimate question: if interventions based on measurements have any impact on long-term outcomes.

Future developments

Randomized controlled intervention trials with long-term neurodevelopmental outcomes need to establish whether perioperative cerebral NIRS monitoring provides benefits over conventional monitoring. These trials will need to delineate clearly what events on cerebral NIRS monitoring will be acted upon, and the treatment response should also be protocolized, to eliminate confounding through different definitions of events or divergent treatment responses. A particular challenge is the determination of appropriate rSO2 thresholds that warrant intervention considering ‘normal’ values may be different within different pediatric surgery populations [28]. Along these lines, it has been suggested to act on a change in baseline within a patient as opposed to an absolute rSO2 threshold [29]. Outside the surgery specific context, cerebral NIRS monitoring has been studied in such a fashion in the neonatal intensive care unit in extremely premature infants in a phase II trial [1], with an ongoing phase III trial [30] illustrating that pediatric randomized controlled intervention trials on NIRS monitoring are feasible. Outside the pediatric context, cerebral NIRS monitoring and goal-directed management of adult patients undergoing coronary artery bypass grafting have been shown to reduce major organ morbidity and overall mortality in a randomized controlled trial [31].

Most of the research to date on NIRS has focused on cerebral monitoring. However, recent data support the notion that changes in NIRS measures from other body sites such as the kidney or muscle (or the ratio of cerebral NIRS to somatic NIRS signals) correlate significantly with systemic indices of oxygenation [32]. Future research may help clarify the role of measurements on other sites of the body for perioperative NIRS monitoring. Key points relating to NIRS monitoring in pediatric anesthesiology are summarized in Table 1.

**Table 1:** Summary of pros and cons of near-infrared spectroscopy (NIRS) in pediatric anesthesiology

Pros	Cons
-Non-invasive and accurate measurement of cerebral oxygenation	-Intraoperative and early postoperative measurements have only a modest association with cognitive, language, and motor skills on long-term follow-up
-Perioperative NIRS measurements are associated with cognitive, language, and motor skills on long-term follow-up	-Using NIRS values alone can lead to overtreatment
-Has the potential for goal-directed therapy when proper absolute or within-patient change thresholds are available	-Current evidence is based on observational studies without a standardized treatment protocol based on NIRS measurements
	-Cost of NIRS devices may not be justified without evidence supporting their use ($10.000 - $20.000)

## Conclusions

NIRS monitoring presents with several pros and cons for its use in pediatric anesthesiology. Due to its current drawbacks, there is no definitive evidence to state it is effective in improving postoperative short and long-term outcomes. Nevertheless, the physiology underlying the measurement, combined with reported associations between NIRS events and neurodevelopmental outcomes, provides a strong starting point for prospective trials evaluating goal-directed therapy regarding brain hypoxia-ischemia.

## References

[1] Hyttel-Sorensen S, Pellicer A, Alderliesten T (2015). Cerebral near infrared spectroscopy oximetry in extremely preterm infants: phase II randomised clinical trial. BMJ.

[2] Mebius MJ, Kooi EMW, Bilardo CM, Bos AF (2017). Brain injury and neurodevelopmental outcome in congenital heart disease: a systematic review. Pediatrics.

[3] Olbrecht VA, Skowno J, Marchesini V (2018). An international, multicenter, observational study of cerebral oxygenation during infant and neonatal anesthesia. Anesthesiology.

[4] Razlevice I, Rugyte DC, Strumylaite L, Macas A (2016). Assessment of risk factors for cerebral oxygen desaturation during neonatal and infant general anesthesia: an observational, prospective study. BMC Anesthesiol.

[5] Gervain J, Mehler J, Werker JF (2011). Near-infrared spectroscopy: a report from the McDonnell Infant Methodology Consortium. Dev Cogn Neurosci.

[6] Dix LM, van Bel F, Baerts W, Lemmers PM (2013). Comparing near-infrared spectroscopy devices and their sensors for monitoring regional cerebral oxygen saturation in the neonate. Pediatr Res.

[7] Sun X, Ellis J, Corso PJ, Hill PC, Chen F, Lindsay J (2015). Skin pigmentation interferes with the clinical measurement of regional cerebral oxygen saturation. Br J Anaesth.

[8] Garvey AA, Dempsey EM (2018). Applications of near infrared spectroscopy in the neonate. Curr Opin Pediatr.

[9] Rodriguez MJ, Corredera A, Martinez-Orgado J, Arruza L (2021). Interference between cerebral NIRS and conjugated billirubin in extremely low birthweight neonates [Article in Spanish]. An Pediatr (Barc).

[10] Madsen PL, Skak C, Rasmussen A, Secher NH (2000). Interference of cerebral near-infrared oximetry in patients with icterus. Anesth Analg.

[11] Busija DW, Heistad DD (1984). Factors involved in the physiological regulation of the cerebral circulation. Rev Physiol Biochem Pharmacol.

[12] Rhee CJ, da Costa CS, Austin T, Brady KM, Czosnyka M, Lee JK (2018). Neonatal cerebrovascular autoregulation. Pediatr Res.

[13] Moritz S, Kasprzak P, Arlt M, Taeger K, Metz C (2007). Accuracy of cerebral monitoring in detecting cerebral ischemia during carotid endarterectomy: a comparison of transcranial Doppler sonography, near-infrared spectroscopy, stump pressure, and somatosensory evoked potentials. Anesthesiology.

[14] Abdul-Khaliq H, Troitzsch D, Schubert S (2002). Cerebral oxygen monitoring during neonatal cardiopulmonary bypass and deep hypothermic circulatory arrest. J Thorac Cardiovasc Surg.

[15] Abdul-Khaliq H, Troitzsch D, Berger F, Lange PE (2000). Regional transcranial oximetry with near infrared spectroscopy (NIRS) in comparison with measuring oxygen saturation in the jugular bulb in infants and children for monitoring cerebral oxygenation [Article in German]. Biomed Eng-Biomed Te.

[16] Altun D, Doğan A, Arnaz A, Yüksek A, Yalçinbaş YK, Türköz R, Sarioğlu T (2020). Noninvasive monitoring of central venous oxygen saturation by jugular transcutaneous near-infrared spectroscopy in pediatric patients undergoing congenital cardiac surgery. Turk J Med Sci.

[17] Thomas L, Flores S, Wong J, Loomba R (2019). Acute effects of hypoxic gas admixtures on pulmonary blood flow and regional oxygenation in children awaiting Norwood palliation. Cureus.

[18] Kussman BD, Laussen PC, Benni PB, McGowan FX, McElhinney DB (2017). Cerebral oxygen saturation in children with congenital heart disease and chronic hypoxemia. Anesth Analg.

[19] Hoffman GM, Brosig CL, Mussatto KA, Tweddell JS, Ghanayem NS (2013). Perioperative cerebral oxygen saturation in neonates with hypoplastic left heart syndrome and childhood neurodevelopmental outcome. J Thorac Cardiovasc Surg.

[20] Spaeder MC, Klugman D, Skurow-Todd K, Glass P, Jonas RA, Donofrio MT (2017). Perioperative near-infrared spectroscopy monitoring in neonates with congenital heart disease: relationship of cerebral tissue oxygenation index variability with neurodevelopmental outcome. Pediatr Crit Care Med.

[21] Del Rosario C, Slevin M, Molloy EJ, Quigley J, Nixon E (2020). How to use the Bayley Scales of Infant and Toddler Development. Arch Dis Child Educ Pract Ed.

[22] Austin EH, Edmonds HL, Auden SM (1997). Benefit of neurophysiologic monitoring for pediatric cardiac surgery. J Thorac Cardiovasc Surg.

[23] Mille T, Tachimiri ME, Klersy C (2004). Near infrared spectroscopy monitoring during carotid endarterectomy: which threshold value is critical?. Eur J Vasc Endovasc Surg.

[24] Kussman BD, Wypij D, Laussen PC (2010). Relationship of intraoperative cerebral oxygen saturation to neurodevelopmental outcome and brain magnetic resonance imaging at 1 year of age in infants undergoing biventricular repair. Circulation.

[25] Cholette JM, Rubenstein JS, Alfieris GM, Powers KS, Eaton M, Lerner NB (2011). Children with single-ventricle physiology do not benefit from higher hemoglobin levels post cavopulmonary connection: results of a prospective, randomized, controlled trial of a restrictive versus liberal red-cell transfusion strategy. Pediatr Crit Care Med.

[26] Alfirevic Z, Devane D, Gyte GM, Cuthbert A (2017). Continuous cardiotocography (CTG) as a form of electronic fetal monitoring (EFM) for fetal assessment during labour. Cochrane Database Syst Rev.

[27] Pedersen T, Nicholson A, Hovhannisyan K, Moller AM, Smith AF, Lewis SR (2014). Pulse oximetry for perioperative monitoring. Cochrane Database Syst Rev.

[28] Korcek P, Stranak Z, Sirc J, Naulaers G (2017). The role of near-infrared spectroscopy monitoring in preterm infants. J Perinatol.

[29] Hirsch JC, Charpie JR, Ohye RG, Gurney JG (2009). Near-infrared spectroscopy: what we know and what we need to know--a systematic review of the congenital heart disease literature. J Thorac Cardiovasc Surg.

[30] Hansen ML, Pellicer A, Gluud C (2019). Cerebral near-infrared spectroscopy monitoring versus treatment as usual for extremely preterm infants: a protocol for the SafeBoosC Randomised Clinical Phase III Trial. Trials.

[31] Murkin JM, Adams SJ, Novick RJ (2007). Monitoring brain oxygen saturation during coronary bypass surgery: a randomized, prospective study. Anesth Analg.

[32] Harer MW, Chock VY (2020). Renal tissue oxygenation monitoring—an opportunity to improve kidney outcomes in the vulnerable neonatal population. Front Pediatr.

